# Predictive value of C-reactive protein for progression from prehypertension to hypertension: a prospective cohort study based on the CHARLS database

**DOI:** 10.1186/s12872-026-06375-7

**Published:** 2026-08-05

**Authors:** Yadong Fan, Chao Zhang, Yulan Qian, Zijie Zhu, Xue Tian, Xuechen Cui, Mengxin Wang, Daocheng Wang, Gang Xue

**Affiliations:** 1 Faculty of Medicine, School of Traditional Chinese Medicine, Yangzhou University, No. 136, Jiangyang Middle Road, Yangzhou, 225009 PR China; 2 Department of Cardiology, Yangzhou Hospital Affiliated to Nanjing University of Chinese Medicine, No. 577, Wenchang Middle Road, Yangzhou, 225000 PR China

**Keywords:** C-reactive protein, Prehypertension, Risk assessment, Hypertension, CHARLS database

## Abstract

**Background:**

The key to preventing and treating hypertension is predicting the progression of prehypertension and implementing preventive and control measures. Whether C-reactive protein (CRP) can predict the progression from prehypertension to hypertension remains to be confirmed.

**Methods:**

Based on prospective data from CHARLS from 2011 to 2015, 1,236 middle-aged and elderly individuals with prehypertension were enrolled and stratified into three groups according to their baseline CRP levels using predefined CRP categories. We employed interval-censored Cox models and restricted cubic spline analysis to assess the association between CRP level and hypertension risk, as well as model improvement metrics after incorporating CRP.

**Results:**

During the 4-year follow-up period, 269 participants developed incident hypertension, accounting for 21.8% of the study population. In the adjusted interval-censored Cox model, higher log-transformed CRP was associated with a higher risk of hypertension (HR = 1.34; 95% CI: 1.07–1.68). Because the restricted cubic spline analysis suggested a nonlinear pattern (P for nonlinearity = 0.040), this estimate should be interpreted as an overall summary rather than a constant risk increase across the full CRP range. In the analysis based on clinical CRP categories, participants with CRP > 3 mg/L had a higher risk than those with CRP < 1 mg/L (HR = 1.61; 95% CI: 1.15–2.26). Subgroup analyses showed no significant interactions across age, sex, BMI, systolic blood pressure, or remnant cholesterol groups. After adding CRP to the conventional risk model, the C-index increased from 0.626 to 0.634.

**Conclusions:**

Elevated CRP levels were associated with the progression from prehypertension to hypertension among middle-aged and elderly adults. Adding CRP to conventional risk factors provided modest additional predictive information. CRP may serve as a supplementary indicator for identifying individuals who need closer follow-up, but it should not be used alone for risk assessment.

**Supplementary Information:**

The online version contains supplementary material available at https://doi.org/10.1186/s12872-026-06375-7.

## Introduction

Blood pressure levels with systolic value of 120–139 mmHg and/or diastolic values of 80–89 mmHg are generally defined as prehypertension, an early warning stage associated with an increased likelihood of developing clinical hypertension and related cardiovascular and cerebrovascular diseases [1]. Its global prevalence is increasing, with estimates of 27.98% in Latin America [2], and 33.8% in West Africa [3]. Approximately 20%–30% of patients with prehypertension progress to hypertension within four years [4]. However, traditional risk factors are not always sufficient to identify individuals at higher risk of progressing from prehypertension to clinical hypertension. Simple and accessible biomarkers for this early risk assessment remain limited.

Recent studies have suggested that chronic low-grade inflammation may be one of the mechanisms involved in the development of hypertension [5]. Experimental evidence has indicated that CRP may contribute to endothelial dysfunction by impairing endothelial nitric oxide synthase [6], while pro-inflammatory mediators such as IL-6 promote vascular remodeling via the NF-κB signaling cascade [7]. Recent population-based evidence has also supported the relationship between inflammation-related indicators and hypertension. Mao et al. reported that the advanced lung cancer inflammation index, a composite indicator integrating body mass index, serum albumin, and the neutrophil-to-lymphocyte ratio, was associated with prevalent hypertension and showed a nonlinear relationship [8]. In addition, recent evidence has indicated that activation of the renin–angiotensin–aldosterone system may interact with inflammatory responses, oxidative stress, endothelial dysfunction, and vascular remodeling during the development of cardiovascular and cerebrovascular diseases [9]. In 2025, the American College of Cardiology emphasized the clinical importance of residual inflammatory risk in cardiovascular disease prevention and risk assessment [10]. Once hypertension is established, patients often become trapped in a vicious cycle of metabolic disturbances and target-organ damage, accelerating atherosclerosis and impairing vital organs [11].

CRP reflects systemic inflammatory status [12]and has been positively associated with triglyceride levels [13] and blood pressure [14], but is inversely associated with high-density lipoprotein cholesterol [15]. Metabolic abnormalities also frequently coexist with hypertension. A recent study in a Chinese hypertensive population identified BMI, fasting blood glucose, and smoking status as important factors associated with dyslipidemia [16]. Serum high-sensitivity C-reactive protein provides prognostic information on cardiovascular risk comparable to that of blood pressure and cholesterol measurements [17]. However, the ability of CRP to predict the progression from prehypertension to overt hypertension remains unclear. Therefore, using data from CHARLS, the present study applied an interval-censored Cox model to evaluate the association between baseline CRP and the progression from prehypertension to hypertension and to assess whether CRP provides additional predictive information beyond conventional risk factors.

## Subjects and methods

### Data sources and research subjects

**Fig. 1:**
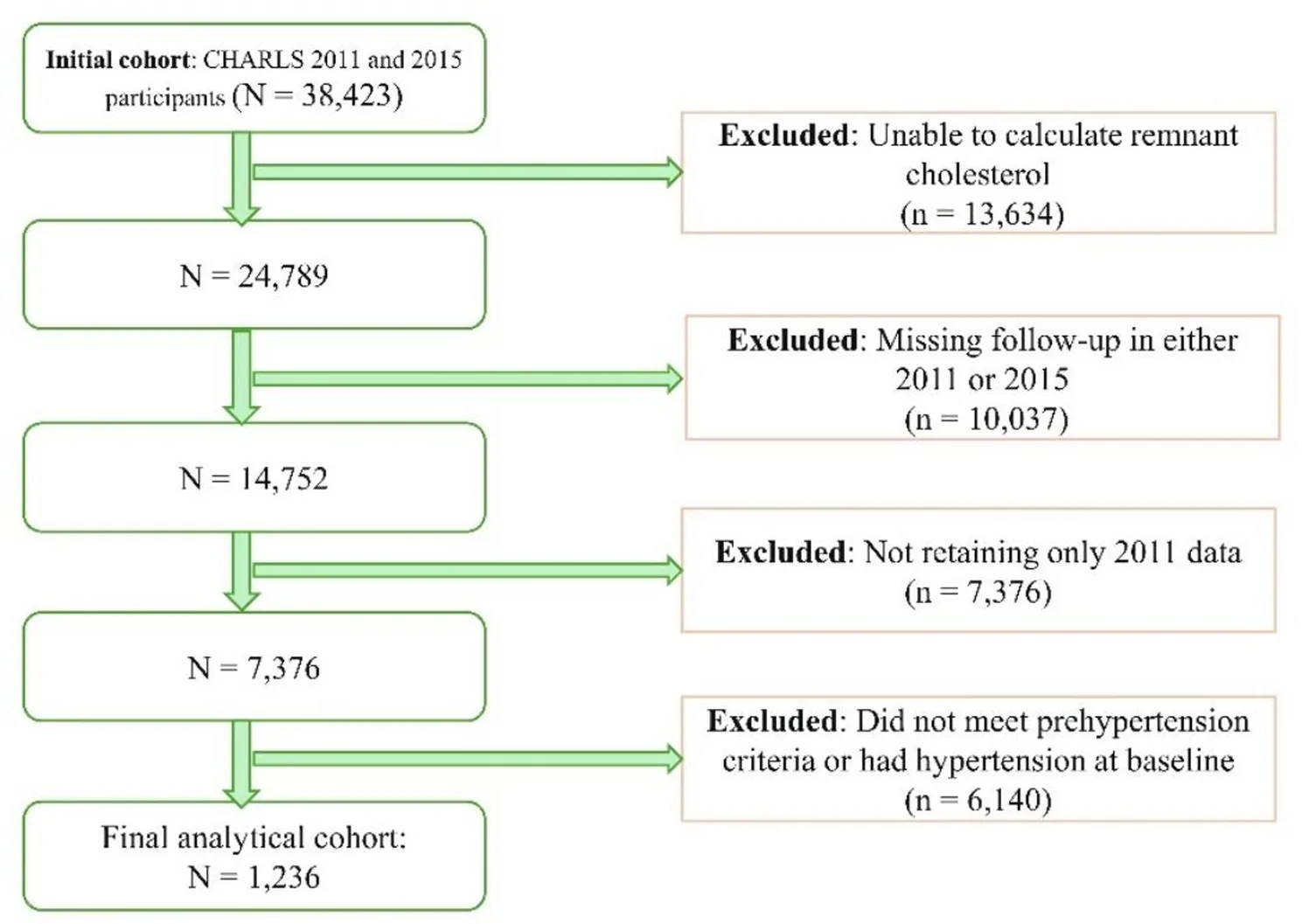
Flowchart of the study process

The China Health and Retirement Longitudinal Study (CHARLS) provided the data for this analysis. This study is a nationally representative survey targeting Chinese adults aged ≥ 45 years, and it was carried out by Peking University [18]. For the present analysis, we used data from the 2011 baseline survey and 2015 follow-up wave of CHARLS. The participant selection process is shown in Fig. 1. Briefly, the merged CHARLS 2011 and 2015 dataset contained 38,423 records. We excluded records without the information needed to calculate remnant cholesterol, followed by those without the required follow-up information across the 2011 and 2015 waves. Records not retained as 2011 baseline observations were then removed. We further excluded individuals who did not meet the baseline prehypertension criteria or who already had hypertension at baseline. The final analytic sample included 1,236 participants.

### Variable definitions

#### Outcome variables

The outcome was whether the participants progressed from prehypertension to clinical hypertension during the 2015 follow-up. Prehypertension was defined as a mean systolic blood pressure of 120–139 mmHg and/or a mean diastolic blood pressure of 80–89 mmHg at baseline [19]. Participants with hypertension at baseline were excluded. This included those with a mean systolic blood pressure ≥ 140 mmHg, a mean diastolic blood pressure ≥ 90 mmHg, self-reported physician-diagnosed hypertension, or current use of antihypertensive medication.

In CHARLS, blood pressure was measured three times using an automated Omron blood pressure monitor. Participants were asked to remain seated, relaxed, and quiet during the measurements. The cuff was placed on the upper arm, and the arm was placed on a flat surface with the upper arm at the level of the heart. The mean of valid systolic and diastolic blood pressure readings was used in the analysis.

We used the clinical diagnostic criteria from the Chinese Guidelines for Hypertension Prevention and Treatment (2024 Revision) [20] as a mean systolic blood pressure ≥ 140 mmHg, a mean diastolic blood pressure ≥ 90 mmHg, self-reported physician-diagnosed hypertension, or current use of antihypertensive medication.

#### Exposure variables

CRP was used as the primary exposure variable in this study. Based on the recommendations of the American Heart Association and Centers for Disease Control and Prevention, the CRP levels were categorized as low (< 1 mg/L), medium (1–3 mg/L), or high (> 3 mg/L). To address the skewed distribution, CRP values underwent natural logarithmic transformation prior to analysis.

#### Covariates

The covariates included five categories: sociodemographic characteristics, lifestyle factors, medical history, anthropometric measurements, and laboratory parameters. Remnant cholesterol was calculated as total cholesterol minus LDL-C minus HDL-C. (Details of the covariates are shown in Table 1).

**Table 1:** Comparison of baseline characteristics among study subjects grouped by baseline c-reactive protein levels

Characteristics	Low CRP (*n*=648)	Medium CRP (*n*=395)	High CRP (*n*=193)	*P* value
Demographic Characteristics				
Age (years)	57.81±8.75	58.24±8.70	59.99±9.13	0.010
Female, *n* (%)	302 (46.6)	215 (54.4)	87 (45.1)	0.026
Married, *n* (%)	581 (89.7)	363 (91.9)	164 (85.0)	0.035
Rural residence, *n* (%)	450 (69.4)	251 (63.5)	128 (66.3)	0.140
Medical History, *n* (%)				
Pulmonary disease	65 (10.0)	31 (7.8)	32 (16.6)	0.006
Arthritis	208 (32.1)	157 (39.7)	67 (34.7)	0.043
Asthma	34 (5.2)	20 (5.1)	23 (11.9)	0.004
Diabetes mellitus	61 (9.4)	50 (12.7)	30 (15.5)	0.040
Lifestyle, *n* (%)				
Ever drank alcohol	245 (37.8)	142 (35.9)	70 (36.3)	0.814
Ever smoked	216 (33.3)	121 (30.6)	68 (35.2)	0.486
Regular physical activity	594 (91.7)	340 (86.1)	164 (85.0)	0.004
Anthropometric Measurements				
Systolic blood pressure (mmHg)	126.09±5.05	126.42±4.99	126.22±5.32	0.595
Diastolic blood pressure (mmHg)	74.90±6.84	75.14±6.96	75.61±6.74	0.444
Pulse rate (beats/min)	71.27±10.22	72.43±9.70	73.68±11.02	0.010
Height (m)	1.59±0.08	1.58±0.11	1.59±0.08	0.021
Weight (kg)	58.31±10.22	60.58±10.29	59.65±11.86	0.003
Waist circumference (cm)	82.83±10.63	85.84±13.22	85.28±11.56	<0.001
Laboratory Parameters				
Body mass index (BMI, kg/m²)	22.90±3.30	24.10±3.50	23.50±4.35	0.222
White blood cell count (×10⁹/L)	6.04±1.65	6.42±1.85	6.98±1.97	<0.001
Fasting blood glucose (mg/dL)	106.63±27.56	110.38±40.42	116.06±46.85	0.004
Creatinine (mg/dL)	0.76±0.17	0.77±0.17	0.79±0.19	0.060
Total cholesterol (mg/dL)	192.29±36.01	192.45±40.92	196.69±41.24	0.355
Triglycerides (mg/dL)	96.91[71.68,139.83]	115.93[77.44,178.77]	111.51[74.34,154.88]	<0.001
HDL-C (mg/dL)	53.27±15.81	48.30±15.20	49.06±14.37	<0.001
LDL-C (mg/dL)	116.56±33.33	116.57±36.14	121.77±36.39	0.161
hs-CRP (mg/L)	0.56[0.40,0.74]	1.53[1.25,2.05]	5.18[3.85,8.36]	<0.001
HbA1c (%)	5.20±0.69	5.35±0.92	5.37±1.02	0.003
Uric acid (mg/dL)	4.25±1.17	4.50±1.29	4.59±1.38	<0.001
Cystatin C (mg/L)	0.96±0.21	0.98±0.23	1.06±0.35	<0.001
Remnant cholesterol (mg/dL)	22.47±20.88	27.58±21.30	25.86±22.06	0.001
Educational Level, *n* (%)				
Below primary school	295 (45.5)	183 (46.3)	83 (43.0)	0.666
Primary school	162 (25.0)	84 (21.3)	47 (24.4)	
Middle school	130 (20.1)	83 (21.0)	46 (23.8)	
High school and above	61 (9.4)	45 (11.4)	17 (8.8)	

1. Continuous variables that meet the normal distribution are expressed as “mean ± standard deviation”; those that do not meet normal distribution are expressed as “median [interquartile range]”; categorical variables are expressed as “*n* (%)”

2. Intergroup comparisons were performed using analysis of variance (ANOVA) for continuous variables or χ² test for categorical variables

3. P < 0.05 statistically significant differences; *P* < 0.01 indicates highly significant differences; *P* < 0.001, extremely significant differences

### Statistical analysis

Because the CHARLS database only records whether participants developed hypertension between 2011 and 2015, without documenting the exact time of onset, we fitted an interval-censored Cox model to explore the connection between CRP levels and incident hypertension. This model is specifically suited for situations in which the exact event time is unknown but is known to lie within a given time interval. Accordingly, the conventional Kaplan–Meier curve and log-rank test were not retained in the revised analysis. Instead, the 4-year cumulative incidence of hypertension was calculated across CRP categories, and modified Poisson regression with robust standard errors was further performed as a sensitivity analysis.

For situations where the proportion of missing covariates is less than 10%, we employed multiple imputation to construct 10 complete datasets [21]. The imputation model included all variables used in the main analysis. Each imputed dataset was analyzed separately, and the estimates were then combined using Rubin’s rules.

We compared a traditional risk factor model (including age, sex, baseline blood pressure, smoking, alcohol use, BMI, and diabetes) with an expanded model that also included log-transformed CRP levels. Model performance was assessed using the Akaike Information Criterion (AIC) [22], likelihood ratio test [23], C-statistic [24], Integrated Discrimination Improvement (IDI), and category-free Net Reclassification Improvement (NRI) [24].

To assess potential nonlinearity, restricted cubic spline (RCS) curves with log-transformed CRP were fitted to the fully adjusted model [25]. CRP was transformed as ln(CRP + 1) to deal with zero values. Three knots were set at the 10th, 50th, and 90th percentiles of log-transformed CRP, and the median CRP level was used as the reference. No winsorization was used in the main analysis. We also excluded participants with CRP > 10 mg/L in a sensitivity analysis. Subgroup analyses were stratified by age, sex, BMI, baseline systolic blood pressure, and remnant cholesterol levels, and interactions were evaluated using likelihood ratio tests [26].

R version 4.5.1 was used for all statistical analyses. Interval-censored Cox regression models were fitted using the icenReg package, and missing data were handled by multiple imputation using the mice package. Restricted cubic spline modeling and model performance evaluations were conducted using the rms package. All tests were two-sided. A P value below 0.05 was taken as statistically significant.

## Results

### Baseline characteristics of study participants

The 1,236 participants with prehypertension were categorized into a low-CRP group (*n* = 648), medium-CRP group (*n* = 395), and high-CRP group (*n* = 193) according to their 2011 CRP levels. The baseline characteristics according to the CRP category are summarized in Table 1. Overall, most baseline variables differed significantly across the CRP groups (*P* < 0.05).

Participants in the high-CRP group were older than those in the other two groups (59.99 ± 9.13 vs. 57.81 ± 8.75 years, *P* = 0.010). The high-CRP group also showed a higher prevalence of chronic conditions, including pulmonary disease (16.6%), asthma (11.9%), and diabetes (15.5%), than with the low- and medium-CRP groups.

Regular physical exercise was most prevalent in the low-CRP group (91.7%, *P* = 0.004). Laboratory findings revealed that participants with higher CRP levels had progressively worsening metabolic profiles, with persistently elevated levels of white blood cells, glycated hemoglobin, uric acid, fasting blood glucose, and cystatin C (all *P* < 0.05). In addition, serum LDL-C, triglycerides, and remnant cholesterol were also higher in the high CRP group (all *P* < 0.001), while HDL-C was the lowest in this group (*P* < 0.001). [See Table 1 for detailed baseline data].

### Four-year cumulative incidence of hypertension according to CRP categories

**Fig. 2:**
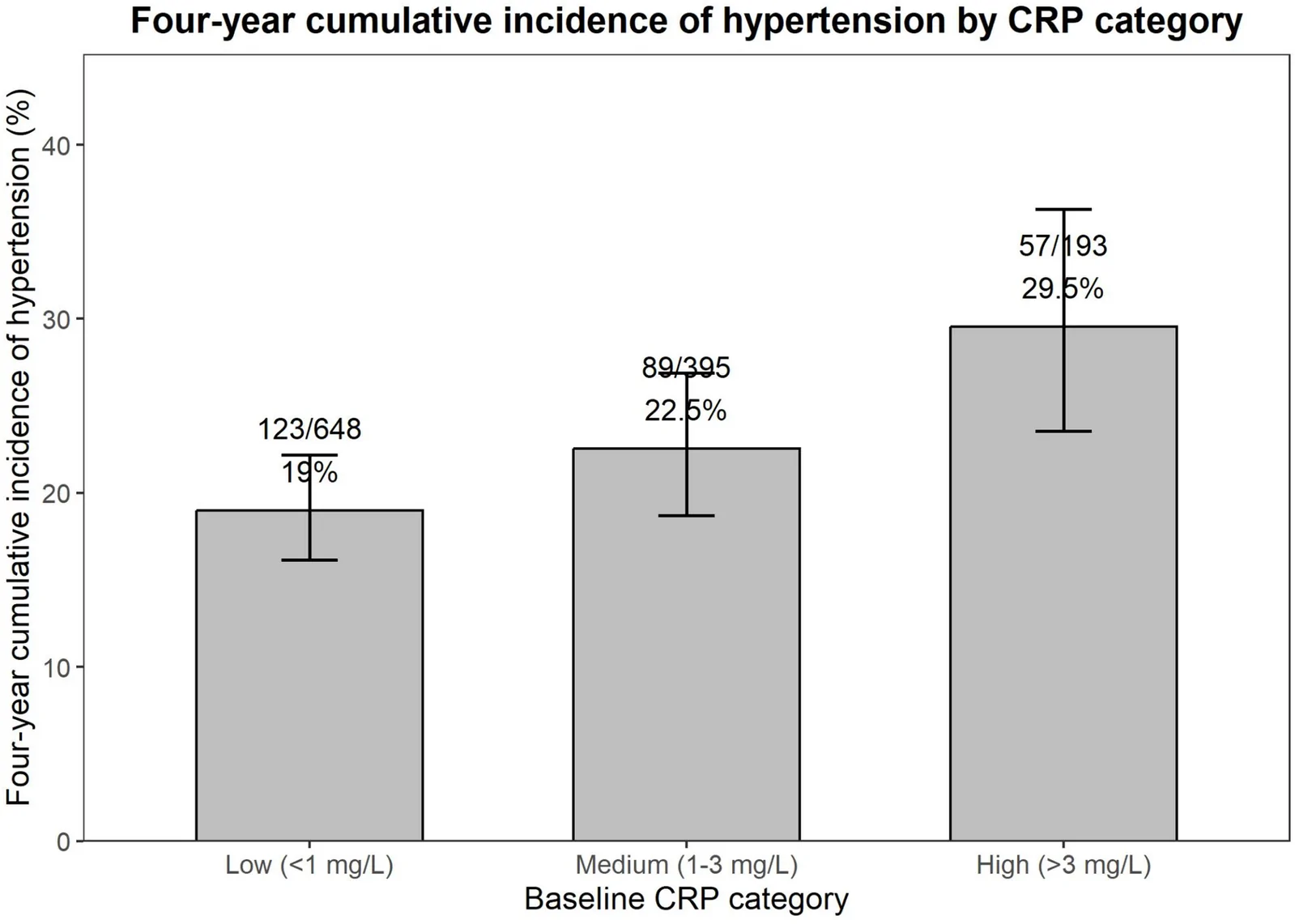
Four-year cumulative incidence of hypertension according to baseline CRP categories. Participants were classified into low CRP (<1 mg/L), medium CRP (1–3 mg/L), and high CRP (>3 mg/L) groups according to baseline CRP levels. The vertical axis shows the cumulative incidence of incident hypertension during the 4-year follow-up period. Error bars indicate 95% confidence intervals

During the 4-year follow-up period, 269 of the 1,236 participants with prehypertension developed incident hypertension. The crude 4-year incidence of hypertension increased across baseline CRP categories, as shown in Fig. 2. Among participants with low CRP, 123 of 648 developed hypertension during follow-up, corresponding to an incidence of 19.0%. The incidence was 22.5% in the medium-CRP group and 29.5% in the high-CRP group. Although this analysis was descriptive, the pattern was in the same direction as the results from the interval-censored Cox model.

### Multivariate cox regression analysis

The adjusted association between CRP and incident hypertension was examined using three interval-censored Cox models with increasing levels of adjustment. Results are presented in Table 2. In the first model, after adjustment for age, sex, and baseline blood pressure in 2011, a 1-unit increase in log-transformed CRP was associated with a 31% higher risk of progression from prehypertension to hypertension. The estimate was almost unchanged after BMI, smoking, drinking, and diabetes were added in Model 2, with the HR remaining at 1.31.This suggests that the association between CRP and hypertension risk was not mainly explained by these lifestyle and traditional risk factors. The fully adjusted Model 3 included lipid variables such as LDL-C and HDL-C. In this model, each 1-unit increase in log-transformed CRP was associated with a 34% higher risk of hypertension (HR = 1.34; 95% CI: 1.07–1.68), suggesting that the association remained after adjustment for traditional risk factors.

**Table 2:** Multivariable Cox regression analysis of the association between CRP and incident hypertension

Model	HR (95% CI)	*P* value
Model 1 (Minimum Adjustment)	1.31(1.05–1.63)	0.017
Model 2 (Core Adjustment)	1.31(1.05–1.63)	0.019
Model 3 (Full Adjustment, Multiple Imputation)	1.34(1.07–1.68)	0.010

HRs represent the association per 1-unit increase in log-transformed CRP

Model 1 was adjusted for age, sex, systolic blood pressure, and diastolic blood pressure

Model 2 was further adjusted for BMI, smoking, drinking, and diabetes

Model 3 was additionally adjusted for LDL-C and HDL-C, with missing covariates handled using multiple imputation

*CRP* C-reactive protein, *HR* hazard ratio, *CI* confidence interval, *BMI* body mass index, *LDL-C* low-density lipoprotein cholesterol, *HDL-C* high-density lipoprotein cholesterol

We further analyzed CRP according to clinical categories. Compared with the low-CRP group, the medium-CRP group showed a nonsignificant trend toward increased risk (HR = 1.24; 95% CI: 0.94–1.65; *P* = 0.132). The high-CRP group had a higher risk of progression to hypertension (HR = 1.61; 95% CI: 1.15–2.26; *P* = 0.005) (Appendix Table 1). Modified Poisson regression showed a similar result, with a higher 4-year cumulative risk in the high-CRP group (RR = 1.50; 95% CI: 1.09–2.07; *P* = 0.003) (Appendix Table 2). In additional analyses, the association was weaker after further adjustment and after excluding inflammatory comorbidities, but the direction was generally unchanged (Appendix Table 3).

### Dose-response relationship between CRP and hypertension risk

**Fig. 3:**
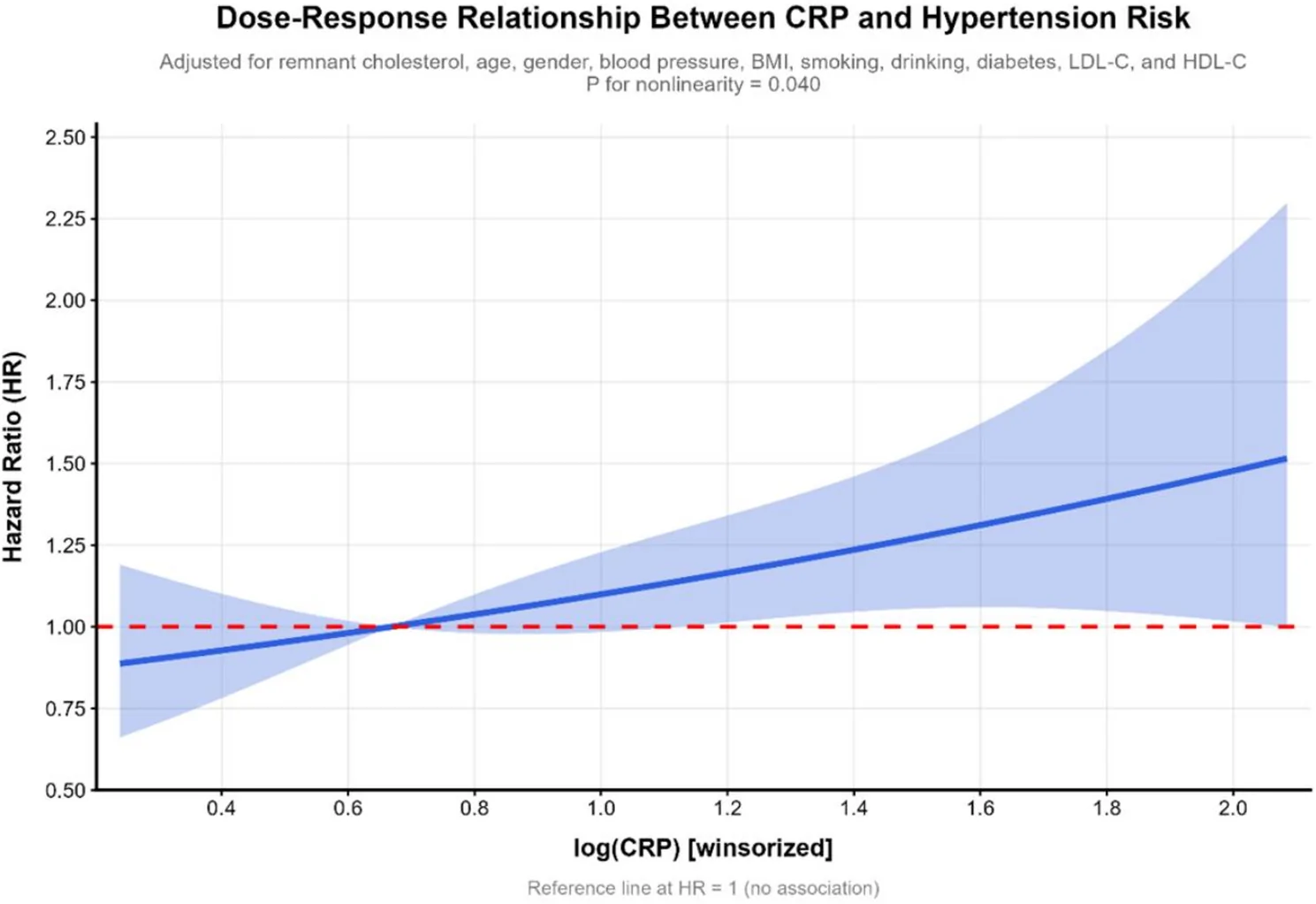
Dose-response relationship between CRP and hypertension risk. Restricted cubic spline fitting was used, adjusting for covariates including age, sex, blood pressure, body mass index, smoking, alcohol consumption, diabetes status, and lipid levels. The vertical axis represents the adjusted hazard ratio (HR), with the reference line set at HR=1.0. The solid line on the curve represents the point estimate, while the shaded area denotes the 95% confidence interval. Nonlinear test *P* = 0.040

Before running the restricted cubic spline analysis, we adjusted for age, sex, baseline blood pressure, smoking, drinking, BMI, LDL-C, HDL-C, remnant cholesterol, and diabetes. Following adjustment, a nonlinear dose-response association was observed between log-transformed CRP and the risk of incident hypertension (Fig. 3). The risk tended to rise with increasing log-transformed CRP. The curve suggested a stronger association at higher CRP levels. However, the confidence intervals were wider in the upper range, so no clear threshold was defined.

### The incremental value of CRP to predictive models

**Fig. 4:**
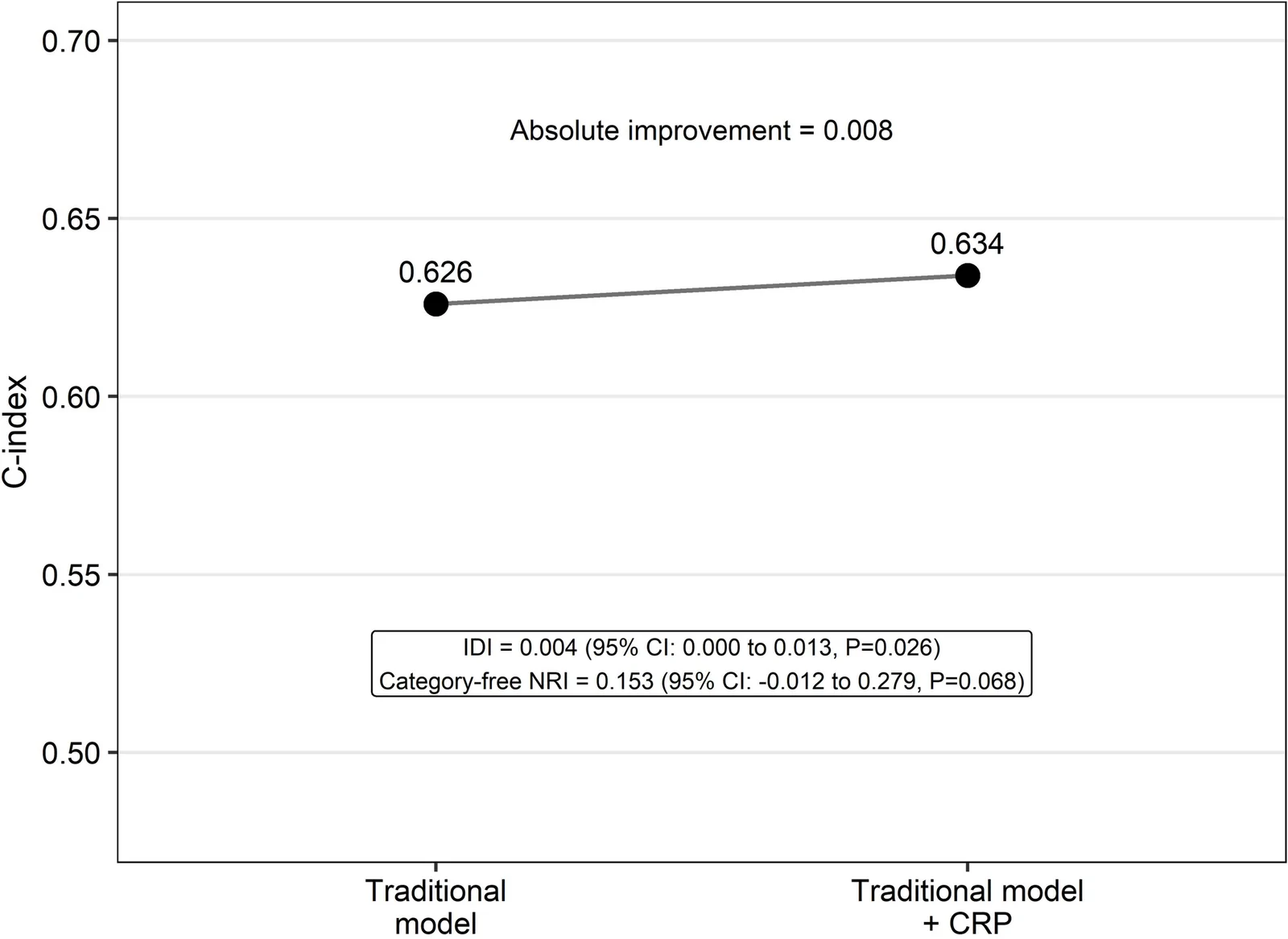
Comparison of model discrimination before and after adding CRP. The traditional model included age, sex, baseline blood pressure, BMI, smoking, drinking, and diabetes. The expanded model further included log-transformed CRP. After adding CRP, the C-index increased from 0.626 to 0.634, with an absolute improvement of 0.008. The IDI was 0.004, and the category-free NRI was 0.153, suggesting a small improvement in predicted risk after CRP was added to the model

We further assessed the incremental value of CRP in hypertension risk prediction. The traditional model included age, sex, baseline blood pressure, BMI, smoking, drinking, and diabetes. When log-transformed CRP was added to the model, the C-index increased from 0.626 to 0.634, with an absolute improvement of 0.008 (Fig. 4). The likelihood ratio test was statistically significant (*P* = 0.013), and the AIC decreased by 4.1 units. The IDI was 0.004 (95% CI: 0.000–0.013, *P* = 0.026), while the category-free NRI was 0.153 (95% CI: -0.012–0.279, *P* = 0.068). These results suggest that CRP added a small amount of predictive information beyond common risk factors. However, the absolute increase in the C-index was below 0.01, and the confidence interval for the category-free NRI crossed the null. Therefore, this statistical improvement should not be interpreted as clear evidence of clinically meaningful risk reclassification. CRP may have limited supplementary value in risk assessment, but it should not be used as a stand-alone clinical prediction marker.

### Subgroup analysis and interactions

We carried out subgroup analyses to further examine how CRP levels relate to the risk of developing hypertension in different populations. Participants were stratified by age, sex, BMI, systolic blood pressure category, and remnant cholesterol level. In most of the resulting subgroups, log-transformed CRP showed a positive relationship with incident hypertension. The interaction tests did not show statistically significant differences across subgroups. Because of this, the variations seen in the subgroup estimates should be read with caution and should not be taken as evidence that the CRP association truly differed by age, sex, BMI, systolic blood pressure, or remnant cholesterol. The number of events was also small in some strata, particularly in the obese subgroup. These subgroup analyses were therefore used only to explore possible patterns, not to define specific high-risk groups in this study (Table 3).

**Table 3:** Subgroup analyses of the association between CRP and incident hypertension

Subgroup	*N*	Events	HR (95% CI)	*P* value	*P* for interaction
Age					0.189
< 55 years	431	77	1.05 (0.70–1.59)	0.799	
55–65 years	558	117	1.41 (1.12–1.79)	0.004	
> 65 years	247	75	0.99 (0.69–1.42)	0.954	
Sex					0.949
Male	632	154	1.21 (0.97–1.51)	0.093	
Female	604	115	1.21 (0.94–1.56)	0.138	
BMI					0.660
Normal weight	733	153	1.24 (1.00–1.55)	0.053	
Overweight	377	78	1.14 (0.83–1.57)	0.407	
Obese	126	38	1.07 (0.63–1.84)	0.793	
SBP					0.114
SBP < 130 mmHg	918	175	1.09 (0.86–1.37)	0.487	
SBP ≥ 130 mmHg	318	94	1.43 (1.09–1.88)	0.010	
Remnant cholesterol					0.368
Low remnant cholesterol	618	127	1.11 (0.85–1.46)	0.443	
High remnant cholesterol	618	142	1.24 (0.99–1.56)	0.061	

Subgroup analyses were performed using interval-censored Cox models. Models were adjusted for age, sex, systolic blood pressure, diastolic blood pressure, and BMI, except for the corresponding stratification variable. HRs represent the association per 1-unit increase in log-transformed CRP. P for interaction was calculated by adding a multiplicative interaction term between log-transformed CRP and each subgroup variable

## Discussion

Using CHARLS data from 2011 to 2015, we found that baseline CRP was related to the later progression from prehypertension to hypertension. The association was still present after adjustment for demographic characteristics, baseline blood pressure, lifestyle factors, diabetes, BMI, and lipid profiles. A similar pattern was seen when CRP was analyzed by clinical categories: participants with CRP > 3 mg/L had a higher risk than those with CRP < 1 mg/L. The spline analysis added another layer to this finding. The curve did not suggest a simple linear increase across the whole CRP range, and the upward trend appeared more evident at higher CRP levels. The confidence intervals were also wider in this part of the curve, so the result needs a cautious reading. When CRP was added to the traditional model, the C-index increased only slightly. Taken together, these findings suggest that CRP may provide some additional information, but it is unlikely to be strong enough as a single predictor for clinical use.

The present findings fit with the growing evidence that inflammation is involved in hypertension. CRP, as a marker of systemic inflammation, has been linked to blood pressure, lipid metabolism, vascular injury, and cardiovascular risk in different populations [27]. Plante et al. found that several inflammatory cytokines, including IL-1β, TNF-α, and IFN-γ, were related to the development of hypertension in the REGARDS cohort [28]. These findings support a simple but important point: hypertension is not only a problem of blood pressure load. Chronic low-grade inflammation may also be involved in its development [29]. Our study extends this line of evidence to people with prehypertension, a group in whom the disease process may have begun but clinical hypertension has not yet been established.

Previous studies have usually examined either normotensive populations or patients with established hypertension. We focused on the stage between the two. This stage is worth studying because blood pressure is already elevated, yet there is still an opportunity to intervene before clinical hypertension develops [4]. In this context, a higher CRP level may indicate a heavier inflammatory burden and a greater tendency to progress over time. The statistical approach also deserves mention. CHARLS recorded hypertension status at follow-up, but not the exact date of onset. For this reason, the interval-censored Cox model fitted the data structure better than a conventional time-to-event model. The modified Poisson analysis and the extended adjusted model gave results in the same direction, although the estimate became weaker after more covariates were included.

Several possible mechanisms may explain the association. Chronic low-grade inflammation can damage endothelial function and reduce nitric oxide bioavailability [30]. As a result, vasodilation may be weakened and vascular tone may increase. Inflammatory mediators may also promote oxidative stress, vascular smooth muscle cell activation, extracellular matrix deposition, and vascular remodeling [27, 28]. Recent studies have further discussed the role of immune activation in hypertension and target-organ injury [29]. These pathways may help explain why participants with higher CRP levels had a greater risk of hypertension progression. However, based on our data, CRP should not be regarded as a direct causal factor. It may also be a signal of a broader inflammatory state.

Metabolic dysfunction may be another part of this process. In our baseline data, participants with higher CRP levels tended to have worse metabolic profiles, including higher glucose-related and lipid-related markers. This suggests that inflammation and metabolic stress may occur together in high-risk individuals. Recent evidence has shown that insulin resistance, reflected by the triglyceride-glucose index, is associated with new-onset hypertension [31]. Other studies have also reported that metabolic dysfunction-associated fatty liver disease is related to hypertension risk, and inflammatory markers may partly mediate this relationship [32]. This evidence agrees with our baseline observations, in which participants with higher CRP levels tended to show more adverse glucose- and lipid-related profiles. Thus, CRP may not represent a single biological pathway, but rather a broader condition in which inflammation and metabolic stress coexist.

The subgroup findings should be interpreted with caution. Although some strata showed numerically stronger estimates, the interaction tests were not statistically significant. This does not support a clear modifying role of age, sex, BMI, baseline systolic blood pressure, or remnant cholesterol. In addition, the number of events was limited in some subgroups, which may have reduced statistical power and increased random variation. We therefore treated these analyses as exploratory and avoided drawing conclusions about specific high-risk subgroups.

In practice, CRP has some appeal because it is simple to measure [10]. For people with prehypertension, it may offer an extra clue when it appears together with higher blood pressure, obesity, diabetes, dyslipidemia, or renal-related abnormalities. These people may need closer follow-up. Still, CRP is not enough for clinical decision-making by itself. In our model, the improvement in discrimination was small after CRP was added. Blood pressure monitoring and routine management of risk factors therefore remain the main part of prevention. Weight control, smoking cessation, a healthy dietary pattern, and regular physical activity are still the basic strategies for reducing the progression from prehypertension to hypertension [33, 34].

This study has several strengths. It used prospective data from CHARLS, a population-based cohort of middle-aged and older adults in China, and focused on people with prehypertension, a clinically important stage before established hypertension. The interval-censored Cox model also matched the follow-up structure of CHARLS, in which the exact date of hypertension onset was unavailable. Restricted cubic splines, subgroup analyses, extended adjustment, and sensitivity analyses were further used to examine the stability of the findings.

There are also limitations to consider. First, CRP was assessed only once at baseline. Since CRP is an acute-phase reactant, its level can be influenced by short-term infection or other transient inflammatory conditions, and one measurement may not represent a participant’s long-term inflammatory status. Such measurement error would probably be non-differential with respect to later hypertension status and could have diluted the observed association, although the present data do not allow us to quantify this effect. Reverse causality is another concern. Some participants with prehypertension may already have had early vascular or hemodynamic changes, which in turn could have contributed to low-grade inflammation. Repeated measurements of CRP, together with longer follow-up, would be helpful for clarifying the temporal sequence.

Residual confounding also remains possible. In addition, the analytic sample represented a selected subset of the CHARLS population. Because CHARLS mainly included middle-aged and older Chinese adults, the findings may not be directly generalizable to younger populations, non-Chinese populations, or cohorts using different blood pressure definitions. The prediction model was not externally validated either. For these reasons, our findings should be viewed as evidence of an association between CRP and hypertension progression, rather than as proof of a causal effect.

## Conclusion

In this prospective cohort study of middle-aged and older adults, elevated baseline CRP was associated with later progression from prehypertension to clinical hypertension. The categorical analysis showed a higher risk among participants with CRP > 3 mg/L than among those with CRP < 1 mg/L. At the same time, the restricted cubic spline analysis suggested that the association was not simply linear across the full CRP range. For this reason, the linear HR should be read as an overall estimate, not as evidence that risk increases by the same amount at every CRP level. After CRP was added to the traditional risk factor model, the improvement in discrimination was small. CRP may therefore offer some supplementary information for risk assessment, but its clinical usefulness still needs further validation.

## Supplementary Information

Supplementary Material 1: Appendix Table 1. Association between clinical CRP categories and incident hypertension in the interval-censored Cox model. Appendix Table 2. Association between clinical CRP categories and 4-year cumulative risk of hypertension in the modified Poisson model. Appendix Table 3. Extended adjusted model for the association between CRP and incident hypertension.

## Data Availability

All data used in this study were obtained from the CHARLS database (https://charls.pku.edu.cn/).

## Abbreviations

CRP: C-reactive protein
CHARLS: China Health and Retirement Longitudinal Study
HR: Hazard ratio
CI: Confidence interval
CVD: Cardiovascular disease
AIC: Akaike Information Criterion
IDI: Integrated Discrimination Improvement
NRI: Net Reclassification Improvement
RCS: Restricted cubic spline
LDL-C: Low-density lipoprotein cholesterol
TG: Triglycerides
HDL-C: High-density lipoprotein cholesterol
BMI: Body mass index
IL-1β: Interleukin-1 beta
TNF-α: Tumor Necrosis Factor-alpha
IFN-γ: Interferon-gamma
