# Supplementary material for: Predictive value of C-reactive protein for progression from prehypertension to hypertension: a prospective cohort study based on the CHARLS database
Source: BMC Cardiovasc Disord. 2026 Aug 5;26:1008. doi: 10.1186/s12872-026-06375-7 (PMC13726066; doi:10.1186/s12872-026-06375-7)
Supplement: Supplementary file 1 [file 12872_2026_6375_MOESM1_ESM.docx]

| Appendix Table 1. Association between clinical CRP categories and incident hypertension in the interval-censored Cox model | | | |
| --- | --- | --- | --- |
| **CRP category** | **HR** | **95% CI** | **P value** |
| Medium CRP vs Low CRP | 1.24 | 0.94–1.65 | 0.132 |
| High CRP vs Low CRP | 1.61 | 1.15–2.26 | 0.005 |

**Table note:** Low CRP was defined as <1 mg/L and was used as the reference group. Medium CRP was defined as 1–3 mg/L, and high CRP was defined as >3 mg/L. HRs were estimated using an interval-censored Cox model. The model was adjusted for age, sex, systolic blood pressure, diastolic blood pressure, BMI, smoking, drinking, diabetes, LDL-C, and HDL-C. CRP, C-reactive protein; HR, hazard ratio; CI, confidence interval; BMI, body mass index; LDL-C, low-density lipoprotein cholesterol; HDL-C, high-density lipoprotein cholesterol.

| Appendix Table 2. Association between clinical CRP categories and 4-year cumulative risk of hypertension in the modified Poisson model | | | |
| --- | --- | --- | --- |
| **CRP category** | **RR** | **95% CI** | **P value** |
| Medium CRP vs Low CRP | 1.20 | 0.91–1.59 | 0.136 |
| High CRP vs Low CRP | 1.50 | 1.09–2.07 | 0.003 |

**Table note:** Low CRP was defined as <1 mg/L and was used as the reference group. Medium CRP was defined as 1–3 mg/L, and high CRP was defined as >3 mg/L. RRs were estimated using modified Poisson regression with robust standard errors to evaluate the 4-year cumulative risk of incident hypertension. The model was adjusted for age, sex, systolic blood pressure, diastolic blood pressure, BMI, smoking, drinking, diabetes, LDL-C, and HDL-C. CRP, C-reactive protein; RR, risk ratio; CI, confidence interval; BMI, body mass index; LDL-C, low-density lipoprotein cholesterol; HDL-C, high-density lipoprotein cholesterol.

| **Appendix Table 3. Extended adjusted model for the association between CRP and incident hypertension** | | | | |
| --- | --- | --- | --- | --- |
| **Analysis** | **N** | **Events** | **HR/RR (95% CI)** | **P value** |
| Extended adjusted model | 1236 | 269 | 1.21 (1.00–1.47) | 0.054 |
| Excluding participants with CRP >10 mg/L | 1195 | 258 | 1.36 (1.06–1.75) | 0.017 |
| Excluding pulmonary disease, asthma, and arthritis | 719 | 168 | 1.23 (0.96–1.57) | 0.109 |

**Table note:** HRs refer to the risk related to each 1-unit increase in log-transformed CRP. The extended adjusted model added waist circumference, physical activity, residence, educational level, cystatin C, fasting glucose, pulmonary disease, asthma, arthritis, and remnant cholesterol to the fully adjusted model. The sensitivity analyses were adjusted for age, sex, systolic blood pressure, diastolic blood pressure, BMI, smoking, drinking, diabetes, LDL-C, and HDL-C. CI, confidence interval; CRP, C-reactive protein; BMI, body mass index; LDL-C, low-density lipoprotein cholesterol; HDL-C, high-density lipoprotein cholesterol.
